# Ferroptosis in Ischemic Stroke and Related Traditional Chinese Medicines

**DOI:** 10.3390/molecules29184359

**Published:** 2024-09-13

**Authors:** Runchen Ma, Xiaohui Sun, Zhaofeng Liu, Jianzhao Zhang, Gangqiang Yang, Jingwei Tian, Yunjie Wang

**Affiliations:** Key Laboratory of Molecular Pharmacology and Drug Evaluation, Ministry of Education, Collaborative Innovation Center of Advanced Drug Delivery System and Biotech Drugs in Universities of Shandong, School of Pharmacy, Yantai University, Yantai 264005, China; celtics5920@163.com (R.M.); sxh163yx2022@163.com (X.S.); m17852656495@163.com (Z.L.); zhangjianzhao@163.com (J.Z.); oceanygq@ytu.edu.cn (G.Y.); tianjingwei618@163.com (J.T.)

**Keywords:** stroke, ferroptosis, traditional Chinese medicine, natural products

## Abstract

Stroke is a severe neurological disorder resulting from the rupture or blockage of blood vessels, leading to significant mortality and disability worldwide. Among the different types of stroke, ischemic stroke (IS) is the most prevalent, accounting for 70–80% of cases. Cell death following IS occurs through various mechanisms, including apoptosis, necrosis, and ferroptosis. Ferroptosis, a recently identified form of regulated cell death characterized by iron overload and lipid peroxidation, was first described by Dixon in 2012. Currently, the only approved pharmacological treatment for IS is recombinant tissue plasminogen activator (rt-PA), which is limited by a narrow therapeutic window and often results in suboptimal outcomes. Recent research has identified several traditional Chinese medicines (TCMs) that can inhibit ferroptosis, thereby mitigating the damage caused by IS. This review provides an overview of stroke, the role of ferroptosis in IS, and the potential of certain TCMs to inhibit ferroptosis and contribute to stroke treatment.

## 1. Introduction

Stroke, also known as cerebral stroke, is a cerebrovascular disease with a high incidence of disability and mortality, often occurring suddenly and unexpectedly [1]. Strokes can be categorized into several subtypes, including ischemic stroke (IS), hemorrhagic stroke, subarachnoid hemorrhage, cerebral venous thrombosis, and spinal cord stroke. Among these, IS is the most prevalent, accounting for 70–80% of all cases [2]. The subtypes of IS are further distributed as follows: atherosclerosis accounts for 23% of cases, small vessel occlusion for approximately 22%, cardioembolism for 22%, and other causes for 29% [3]. IS can also be classified based on the area of cerebral ischemia, as either focal or global stroke, and based on the duration of ischemia, as either transient or permanent cerebral ischemia [4,5].

Generally, in the event of cerebral ischemia, patients may experience symptoms such as sudden loss of balance, blurred or impaired vision, double vision or gaze preference, facial weakness or asymmetry, limb weakness, and speech difficulties. These symptoms arise from brain dysfunction caused by reduced blood flow [6,7]. Currently, during the acute phase of IS, the treatment strategy focuses on optimizing hemodynamics by managing blood volume, blood pressure, blood glucose, and cardiovascular status, while also aiming to restore blood flow to potentially salvageable brain tissue through early reperfusion, either via intravenous thrombolysis or endovascular thrombectomy. Clinical trials suggest that the optimal window for intravenous thrombolysis is within 4.5 h of symptom onset, while the window for endovascular thrombectomy is even narrower, often requiring imaging to assess the viability of restoring damaged tissue [8]. Based on the primarily pathological features of IS, it can be categorized into the infarct core and the surrounding ischemic penumbra. In the infarct core, severe reduction in blood flow leads to cell death and tissue necrosis, while the ischemic penumbra represents a dynamic, less severely affected area where blood flow is reduced but still sufficient to sustain tissue viability. Both pharmacological interventions and endovascular procedures aim to prevent the penumbra from progressing to irreversible infarction by rapidly restoring blood flow [5,9]. However, the damage is not confined to the ischemic phase. Reperfusion, the process of restoring blood flow and oxygenation, can exacerbate tissue injury and significantly impact neuronal function [10]. The mechanisms underlying ischemia–reperfusion injury include excitotoxicity, mitochondrial dysfunction, neuroinflammation, blood–brain barrier (BBB) disruption, and extensive neuron cell death [11]. Consequently, both basic and clinical research efforts have been directed toward mitigating neuronal death caused by ischemia-reperfusion injury and preserving the function of existing neurons.

Since the 1970s, cell death has been broadly categorized into apoptosis, autophagy, and necrosis based on cellular morphology [12]. Ferroptosis, a recently identified form of cell death, is characterized by extensive lipid peroxidation, leading to membrane damage and iron ion-dependent cell necrosis [13]. This concept of ferroptosis as a distinct mode of cell death was first introduced by Dixon in 2012 [14]. The initial discovery of ferroptosis stemmed from observations that nutrient depletion, specifically cystine, led to a unique form of cell death distinguishable from other types by its microscopic morphology. Nota-bly, cell viability could be restored by supplementing with glutathione (GSH) [15,16]. In animal studies, hepatic necrosis induced by vitamin E and cystine deficiency in rats was found to be reversible with selenium-containing compounds [17]. These findings laid the foundation for understanding ferroptosis. The discovery of glutathione peroxidase 4 (GPX4) further clarified the role of selenium-containing compounds, with GPX4 subse-quently recognized as a key regulator in the ferroptosis pathway [18]. Three classical path-ways are now known to be involved in the inhibition of ferroptosis: the system Xc^−^/GPX4 axis, the Ferroptosis suppressor protein 1 (FSP1)/coenzyme Q (CoQ) axis, and the guano-sine triphosphate cyclohydrolase 1 (GCH1)/tetrahydrobiopterin (BH_4_)/dihydrofolate re-ductase (DHFR) axis [17]. A substantial body of research has demonstrated that ferropto-sis plays a significant role in the cell death mechanism associated with IS, making the prevention of cellular ferroptosis a promising avenue for future stroke therapies.

Currently, rt-PA remains one of the few treatment options available during the acute phase of IS. However, its applicability is limited, necessitating the development of more effective therapeutic agents. In recent years, natural products and herbal medicines have garnered increasing attention, with several studies indicating that certain natural products can improve neurological function after cerebral ischemia–reperfusion by reducing fer-roptosis. Therefore, this review summarizes current knowledge on the etiology, classify-cation, and treatment of IS, the potential role of ferroptosis in its therapy, and the natural products and herbs that have demonstrated the ability to mitigate ferroptosis, with the aim of providing a foundation for developing therapeutic strategies for IS.

## 2. Ischemic Stroke

### 2.1. Epidemiology of Stroke

Stroke is a cerebrovascular disease with a high morbidity and significant disability rate, ranking as the second leading cause of death globally and the leading cause of death in China, and it is also recognized as the primary cause of long-term disability in many countries [19,20]. According to the World Health Organization (WHO), a stroke occurs every five seconds worldwide. Approximately 15 million strokes occur each year, with one-third resulting in death and up to 50% of survivors living with disability. As the global population continues to age, the incidence of stroke is expected to rise, with direct medical costs associated with stroke projected to triple between 2012 and 2030, primarily due to an increase in cases among individuals aged 65 to 79 [21].

In China, the impact of stroke is particularly alarming. Stroke has become the second leading cause of death among rural residents and the third leading cause of death among urban residents, accounting for 22.94% of deaths in rural areas and 20.61% of deaths in urban areas. Date from the Global Burden of Disease study indicate that between 2010 and 2019, there was no significant change in the stroke mortality rate among urban residents, while the mortality rate among rural residents showed a fluctuating upward trend, sur-passing that of urban residents [1]. The prevalence of IS increased from 1100 per 100,000 to 1256 per 100,000 during this period, reflecting an overall upward trend, while the prev-alence of hemorrhagic stroke decreased from 232 per 100,000 to 215 per 100,000 [1]. In 2019, there were 3.94 million new stroke cases in China, with stroke-related mortality rates of 174 per 100,000 men and 133 per 100,000 women, indicating a significantly higher mortal-ity rate in men compared to women [4].

### 2.2. Etiology and Pathogenesis of Ischemic Stroke

IS can be classified into several subtypes based on etiology: large artery atheroscle-rotic infarction, cardiogenic embolism, small artery occlusion infarction, infarction due to other specific causes, and cryptogenic stroke. Approximately 23% of ISs are attributed to large artery atherosclerosis, which often results from the rupture of atherosclerotic plaques. This rupture can lead to in situ thrombosis and distal embolization, with the rup-ture of carotid artery plaques commonly causing significant platelet activation. Addition-ally, less common causes of IS include the narrowing of the vertebrobasilar or intracranial arteries [22].

The pathological damage in IS is influenced by several mechanisms, primarily vas-cular obstruction, thromboembolism, and insufficient cerebral perfusion. Vascular ob-struction and thromboembolism are the leading causes of IS, and an effective treatment strategy involves the prompt recanalization of blood vessels to reduce the duration of cer-ebral hypoxia and ischemia. It is well established that insufficient blood supply to brain tissue initially leads to a reversible loss of tissue function, and prolonged ischemia results in irreversible neuronal death and infarction. During ischemia, electrical function is lost, calcium ions flow inward, membrane function becomes compromised, and calcium-de-pendent excitotoxicity triggers a cascade of cellular damage [8].

**Figure 1:**
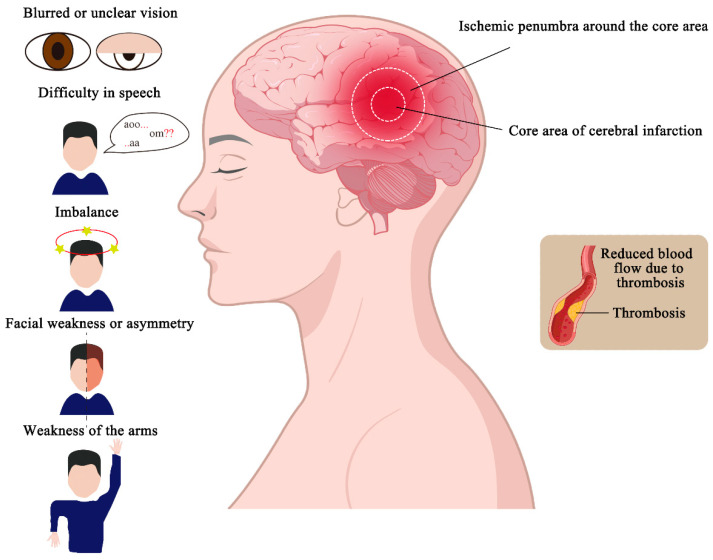
Partitioning and symptoms of ischemic stroke. Ischemic strokes are caused by the for-mation of blood clots in the blood vessels, which reduces blood flow and causes infarction in some areas. The infarcted area can be divided into the core area of the cerebral infarction and the ischemic penumbra around the core area. And ischemic strokes cause abnormal behavior, such as a sudden onset of imbalance, blurred or unclear vision, facial weakness or asymmetry, weakness of the arms or legs, difficulty in speech, and so on.

The lesion area in IS typically consists of a core infarct area and the surrounding is-chemic penumbra. The core infarct area, which contains a large amount of necrotic tissue, represents irreversible damage. Surrounding the core is the ischemic penumbra, a region with less severe blood flow reduction that remains dynamic and unstable. If blood flow is reperfused within a certain time frame, neurons in the penumbra can be salvaged, and their function restored. The extent and severity of the infarcted area increase over time, so minimizing the duration of hypoxia and ischemia is crucial for reducing the size of the core infarct, restoring function in the penumbra, and improving patient outcomes (Figure 1) [5].

### 2.3. Clinical Manifestations and Treatment of Ischemic Stroke

The clinical manifestations of cerebral infarction primarily depend on the size and location of the infarct, with symptoms closely related to the distribution of the affected vessels. Common symptoms of IS include a sudden onset of imbalance, blurred or unclear vision, double vision or gaze preference, facial weakness or asymmetry, weakness of the arms or legs, and difficulty in speech (Figure 1) [6]. These symptoms result from tempo-rary cerebral dysfunction due to reduced blood flow and may be accompanied by hyper-tension, diabetes mellitus, and cardiac arrhythmias, in addition to neurological deficits caused by vascular occlusion [19]. In clinical practice, stroke diagnosis is often supported by the use of biomarkers, which may include molecules present in body fluids (such as blood, cerebrospinal fluid, or urine) or physical measurements from tissues. Molecular biomarkers include proteins, metabolites, lipids, and ribonucleic acids (RNAs), and their combined use can enhance stroke detection and improve diagnostic accuracy [23].

The treatment of IS requires a tailored approach based on the etiology, pathogenesis, and timing of onset. In the acute phase of IS, treatment typically involves restoring blood flow through pharmacological thrombolysis or mechanical thrombectomy. Studies from the 1990s demonstrated that patients treated with intravenous rt-PA showed better recov-ery and lower Modified Rankin Scale (mRS) scores compared to those receiving placebo. Intravenous thrombolysis was found to be most effective within the 0–3 h and 3–4.5 h time windows [7,24]. For patients with large vessel occlusions, mechanical thrombectomy is commonly performed using stent retrievers or suction catheters. These devices are intro-duced through the femoral or radial artery via a guiding catheter and advanced to the occluded cerebral artery under fluoroscopic angiographic guidance. Stent retrievers are wire meshes that expand at the site of the occlusion, capturing the clot for removal along with the stent. Suction devices, on the other hand, use proximal suction to aspirate the clot from the occluded artery [7]. The clinical management of IS remains focused on interven-tions to restore blood flow, either through pharmacological thrombolysis or mechanical clot removal [25]. However, due to the narrow therapeutic window and contraindications to thrombolysis, only approximately 11% of IS patients are eligible for rt-PA treatment.

## 3. Ferroptosis and Ischemic Stroke

Cell death occurs regularly in normal tissues and is essential for maintaining tissue function and morphology [26]. Ferroptosis, a novel form of programmed cell death, was first identified by Dixon et al. in 2012. This process is iron-dependent and is biochemically characterized by the accumulation of iron and lipid reactive oxygen species (ROS), the depletion of GSH, and the oxidation of nicotinamide adenine dinucleotide phosphate (NADPH) [14].

The morphology of cells undergoing ferroptosis differs significantly from that seen in other forms of cell death. Unlike necrosis, which is characterized by cell swelling, the rupture of cell membranes, and organelle disintegration, and unlike apoptosis, which involves cellular shrinkage and chromatin condensation, ferroptosis cells exhibit distinct features: a normal nucleus, wrinkled mitochondria with increased membrane density, and reduced or absent mitochondrial cristae [27]. Ferroptosis plays a crucial regulatory role in the pathogenesis of various diseases, including tumors, neurological disorders, acute kid-ney injury, and ischemia–reperfusion injury [26]. Numerous studies have highlighted fer-roptosis as a significant mechanism of cell death in IS, underscoring its importance in stroke pathophysiology and suggesting that targeting ferroptosis could be a promising therapeutic strategy in the future.

Maintaining iron homeostasis is particularly vital for the brain’s normal physiological function. Under normal conditions, the BBB protects the brain from fluctuations in iron levels. However, during IS, the integrity of the BBB is compromised, disrupting iron ho-meostasis. This disruption allows free iron ions and ferritin to enter the brain parenchyma, ultimately leading to the induction of ferroptosis [28].

### 3.1. Indicators of Ferroptosis

#### 3.1.1. Accumulation of Lipid Peroxides

Fatty acids can generally be categorized into three types: saturated fatty acids, monounsaturated fatty acids, and polyunsaturated fatty acids (PUFAs). Among the various cell membrane lipids that can undergo oxidation, the oxidation of PUFAs—resulting in the formation of lipid peroxides—plays a crucial role in the process of ferroptosis [29]. Lipid peroxidation typically occurs in two stages. The initial stage produces lipid hydrop-eroxides (LOOHs), while the subsequent stage generates secondary products, such as malondialdehyde (MDA) and 4-hydroxynonenal (4-HNE), both of which significantly in-crease during ferroptosis [29].

The accumulation of lipid peroxides is generally driven by two primary factors: the increased production of ROS through the Fenton reaction, which leads to a rise in lipid peroxides, and a series of enzymatic reactions involved in lipid peroxide synthesis [12]. Lipid peroxidation results from the direct interaction of oxidizing agents with the carbon-carbon double bonds in lipids, including phospholipids, glycolipids, and cholesterol [12]. ROS are typically generated as a result of the partial reduction of oxygen and include hydrogen peroxide (H_2_O_2_), superoxide anion (O_2_^−^), and hydroxyl radical (HO^−^). These ROS are produced by various enzymes, such as those in the mitochondrial electron transport chain and NADPH oxidases (NOXs), and are converted to H_2_O_2_ by the enzyme superoxide dismutase (SOD) [12]. In the Fenton reaction-mediated lipid peroxidation, the H_2_O_2_ oxidizes Fe^2+^, leading to the production of Fe^3+^, HO·, and OH^−^. Additionally, O_2_^−^ reacts with Fe^3+^ to regenerate Fe^2+^, a process known as the Haber–Weiss cycle. However, fatty acid β-oxidation in mitochondria can be mitigated by the depletion of fatty acids, thereby reducing lipid peroxidation [12].

The process of lipid peroxidation involves the reaction of an oxidizing agent with hydrogen in a methylene group to link the double bonds in a polyunsaturated fatty acid, forming a lipid radical (L). This lipid radical (L) then reacts with oxygen to form a lipid peroxyl radical (LOO), which extracts a hydrogen atom from another lipid to form a new L- and a LOOH. Acyl-CoA Synthetase Long-Chain Family Member 4 (ACSL4) is a critical factor in lipid peroxidation and ferroptosis. ACSL4 catalyzes the addition of CoA to the long-chain polyunsaturated bonds of arachidonic acid, thereby promoting the esterifica-tion of PUFAs into phospholipids. In the presence of Lysophosphatidylcholine Acyltrans-ferase 3 (LPCAT3), the acyl group is added, playing a role in lipid signaling [30]. Eventu-ally, lipids are oxidized by lipoxygenases. Arachidonate lipoxygenases (ALOXs) have many isoforms, i.e., arachidonate 5-lipoxygenase (ALOX5), arachidonic acid 12-lipoxygenase, type 12S (ALOX12), arachidonic acid 12-lipoxygenase, type 12R (ALOX12B gene), arachidonate 15lipoxygenase (ALOX15), arachidonate 15 -lipoxygenase type B (ALOX15B), and arachidonate lipoxygenase 3 (ALOXE3), and they induce ferroptosis by oxidizing PUFA-phosphatidylethanolamines (PUFA-PE) [31].

#### 3.1.2. Iron Accumulation

Iron is a trace element, one of the more abundant elements in the human body, which is important for maintaining and inducing cell survival and apoptosis, and is an indispen-sable element in the human body. Normal physiological concentrations of iron are essen-tial for the maintenance of deoxyribonucleic acid (DNA), RNA, and protein synthesis, electron transport, cellular respiration, cell differentiation, and proliferation. When prob-lems occur in electron reception and transport, cells undergo oxidative damage and apop-tosis [32,33]. In mammalian cells, iron uptake and transport proteins or receptors transport iron, participate in the production of lipid peroxides mediated by the Fenton reaction, and transport iron to enzymes involved in lipid peroxidation [34].

Transferrin receptor 1 (TFR1) is a membrane protein responsible for transferring Fe^3+^ into the cell. Under normal physiological conditions, most iron binds to ferritin or the transferrin receptor. Ferritin tightly binds iron, with each ferritin molecule capable of binding two Fe^3+^ ions, which are then transferred to transferrin to facilitate the delivery of Fe^3+^ to ferritin. In endosomes, a decrease in ambient pH results in the release of Fe^3+^, which is subsequently reduced to Fe^2+^ by the enzyme STEAP3 metalloreductase. Fe^2+^ then enters the cytoplasm through SLC11A2 (solute carrier family 11 member 2)/DMT1 (divalent metal transporter 1) on the membrane, while ferritin, now free of Fe^3+^, returns to the cell surface to take up more iron [26]. Ferroportin (FPN) is the only intracellular protein re-sponsible for exporting iron ions out of the cell. However, during cerebral ischemia, the ability of FPN to export iron is diminished, leading to further accumulation of iron ions. Fe^2+^ constitutes the majority (80–90%) of the intracellular iron pool and is highly redox-active. Fe^2+^ catalyzes the formation of hydroxyl radicals from H_2_O_2_, which in turn induces the accumulation of lipid peroxides, ultimately leading to ferroptosis [12].

### 3.2. The Classic Pathway of Ferroptosis

Ferroptosis can be triggered by various oxidative stressors, and while the mecha-nisms may differ, the primary pathways can be broadly categorized. These include path-ways dependent on transporters located on the cell membrane (e.g., the regulation of the system Xc^−^ transporter, which controls the influx and efflux of cystine) and pathways de-pendent on intracellular oxidoreductases (e.g., direct action on GPX4 that induces or in-hibits the production of lipid peroxides, thereby modulating ferroptosis) [35]. Both modes of regulation involve multiple organelles, including the mitochondria, endoplasmic retic-ulum, Golgi apparatus, nucleus, and lysosomes, which work together to regulate ferroptosis [36]. This section focuses on elucidating three classical pathways that regulate ferroptosis: the system Xc^−^/GPX4 axis, the FSP1/CoQ axis, and the GCH1/BH_4_/DHFR axis.

#### 3.2.1. System Xc^−^/GPX4 Axis

**Figure 2:**
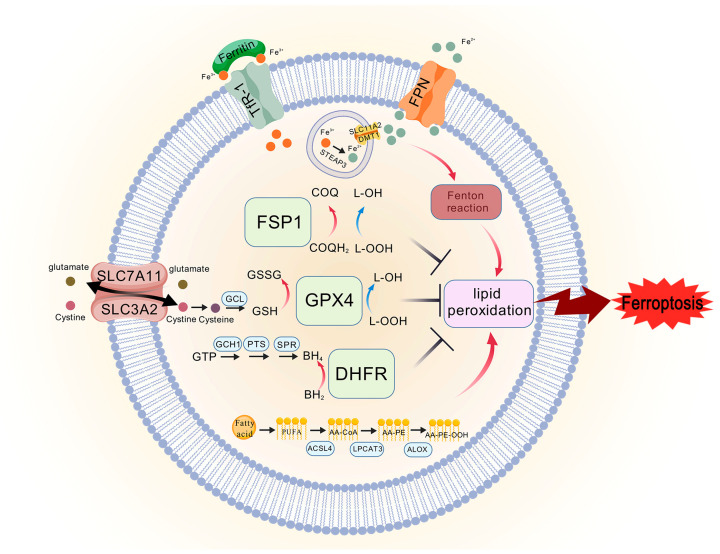
The core mechanisms of ferroptosis. The system Xc^−^/GPX4 axis regulates ferroptosis by modulating cystine uptake, GSH synthesis, and reduction in lipid peroxides by GPX4. The FSP1/CoQ axis is parallel and independent system that synergizes with the GSH-GPX4 axis to in-hibit ferroptosis, and FSP1 reduces CoQ to CoQH_2_, preventing the production of lipid peroxides and inhibiting ferroptosis. The GCH1/BH4/DHFR axis can protect against ferroptosis in a GPX4-independent manner, GCH1-BH4 axis controls the production of the endogenous antioxidant BH4, thereby regulating ferroptosis. TFR1 transports Fe^3+^ into the membrane, and in the endosomes, STEAP3 reduces Fe^3+^ to Fe^2+^, and Fe^2+^ enters the cytoplasm via SLC11A2/DMT1. When cerebral is-chemia stroke occurs, the ability of FPN to transport iron ions outward is reduced, which exacer-bates the deposition of iron, and Fe^2+^ participates in the Fenton reaction, which induces the accumu-lation of lipid peroxides, leading to ferroptosis. PUFA exacerbates lipid peroxidation in the presence of ACSL4, LPCAT3, and ALOX.

System Xc^−^ is a cystine-glutamate antiporter that plays a crucial role in the cell’s an-tioxidant defense. It consists of a catalytic subunit, xCT (Solute Carrier Family 7 Member 11, SLC7A11), and a regulatory subunit, 4F2 (4F2hc)/Solute Carrier Family 3 Member 2 (SLC3A2), linked by a disulfide bond, and is widely distributed in phospholipid bilayers [37]. System Xc^−^ facilitates the exchange of glutamate and cystine in equal proportions across the cell membrane. Once inside the cell, cystine is reduced to cysteine, which is a critical precursor in the synthesis of reduced GSH [14,37]. GSH, a tripeptide containing a γ-amide bond and a sulfhydryl group, is synthesized from three amino acids by the en-zyme glutamate-cysteine ligase (GCL), and serves as a necessary cofactor for the synthesis of GPX4 a cofactor necessary for the synthesis of GPX4 [12]. The Glutathione peroxidase (GPX) family includes several isoforms that function in different cells and tissues. Unlike other GPXs, GPX4 reduces intracellular lipid peroxides (L-OOH) to non-toxic lipid alco-hols (L-OH) while oxidizing GSH to glutathione disulfide (GSSG) [12,38]. The inhibition of system Xc^−^ leads to cysteine deficiency in the cell, and since sufficient cysteine is re-quired for GPX4 synthesis, its inhibition results in decreased GPX4 production. This re-duction in GPX4 impairs the cell’s ability to clear lipid peroxides, ultimately leading to ferroptosis (Figure 2) [39]. A study by Hideyo Sato demonstrated that a redox imbalance occurs in mice deficient in the glutamate/cystine transporter. Although xCT^−/−^ mice appear healthy, their plasma GSH levels are significantly lower compared to xCT^+/+^ mice, and embryonic fibroblasts derived from xCT^−/−^ mice do not survive in conventional media un-less supplemented with beta-mercaptoethanol (β-ME) or vitamin E [40].

#### 3.2.2. FSP1/CoQ Axis

Coenzyme Q, also known as ubiquinone, is a 2-methyl-1,3-butadiene benzoquinone commonly found in eukaryotes. It is a lipophilic molecule with redox activity, characterized by a redox-active benzoquinone head group and polyisoprenoid side chains, which allow it to be embedded within lipid-rich structures. CoQ is located within the plasma membrane and various endomembrane systems, where it plays a crucial role in mitochon-drial energy production and ROS generation. In the mitochondrial respiratory chain, CoQ functions as an electron carrier, transferring electrons from complexes I and II to complex III. CoQ exists in three redox states: the fully oxidized state (CoQ), the partially reduced state (CoQH·), and the fully reduced state (CoQH_2_). The transitions between these forms are integral to the respiratory chain’s function [41]. In most cellular membranes, except the plasma membrane, CoQ predominantly exists in its reduced form (70–80% of the total), whereas in the plasma membrane, the reduced form constitutes about 30% [42]. The re-duced form of CoQ, also known as panthenol (CoQ10H_2_), is a potent lipophilic antioxidant. In complex I of the respiratory chain, two electrons from nicotinamide adenine dinucleo-tide (NADH) are transferred to flavin mononucleotide (FMN), then to the iron-sulfur clus-ters, and finally to CoQ. This process first produces a transient semiquinone intermediate, followed by the formation of fully reduced ubiquinol. Dihydroorotic acid dehydrogenase (DHODH) acts as an oxidoreductase that reduces CoQ to CoQH_2_ at the plasma membrane and inner mitochondrial membrane, thereby preventing lipid peroxidation and inhibiting ferroptosis.

The FSP1/CoQ axis operates as a parallel and independent system that synergizes with the GSH-GPX4 axis to inhibit ferroptosis (Figure 2). A decrease in intracellular CoQ levels leads to increased ROS production in mitochondria, triggering lipid peroxidation and promoting ferroptosis [43]. Using a CRISPR/Cas9 screening system, Kirill Bersuker et al. identified FSP1 as a potent ferroptosis resistance factor, highlighting its role as a key component of the CoQ antioxidant system. The overexpression of FSP1 enhances the reduction of CoQ to CoQH_2_, thereby preventing the formation of lipid peroxides and in-hibiting ferroptosis [44]. Furthermore, the inhibition of the CoQ biosynthesis enzyme (COQ2) with 4-chlorobenzoic acid (4-CBA) significantly reduces intracellular CoQ levels, leading to the loss of FSP1 function. These findings suggest that FSP1 and CoQ operate within the same pathway to inhibit lipid peroxidation and resist ferroptosis.

#### 3.2.3. GCH1/BH4/DHFR Axis

BH_4_ is a powerful small-molecule antioxidant that plays a crucial role in the synthesis of nitric oxide, neurotransmitters, and aromatic amino acids. BH4 is synthesized from guanosine triphosphate (GTP) through a series of enzymatic reactions involving GTP cy-clohydrolase 1 (GCH1), 6-pyruvoyltetrahydropterin synthase (PTS), and sepiapterin re-ductase (SPR). Notably, GCH1 is the rate-limiting enzyme in the formation of BH_4_ [17,45]. Additionally, BH_2_ can be reduced to BH_4_ by DHFR, and the inhibition of DHFR can lead to ferroptosis. It is also noteworthy that cells with a high expression of GCH1 are rich in CoQ10, possibly because BH_4_ promotes CoQ10 synthesis and enhances resistance to ferroptosis by converting phenylalanine to tyrosine, which is further converted into 4-OH-benzoate, a precursor of CoQ10 [46].

A recent study identified the GCH1/BH_4_/DHFR axis as a potent pathway regulating ferroptosis through an overexpression screen using a genome-wide activation library (Figure 2) [45]. Cells expressing GCH1 synthesize BH_4_/BH_2_, leading to lipid remodeling that inhibits ferroptosis by selectively preventing the depletion of phospholipids with two pol-yunsaturated fatty acyl tails [46]. In summary, the GCH1-BH_4_ axis regulates ferroptosis by controlling the production of the endogenous antioxidant BH_4_, maintaining the abun-dance of CoQ10, and preventing the peroxidation of abnormal phospholipids with poly-unsaturated fatty acyl tails [46].

### 3.3. Inducers and Inhibitors of Ferroptosis

#### 3.3.1. Inducers of Ferroptosis

Certain inhibitors of system Xc^−^ can induce ferroptosis. A classic example is erastin, a small molecule that induces ferroptosis by inhibiting system Xc^−^, which consists of the light chain xCT encoded by the SLC7A11 genes and the heavy chain 4F2hc encoded by the SLC3A2 genes [47]. Researchers have shown that when erastin is added to the xCT-4F2hc complex, the chlorophenoxy group at one end of the erastin molecule projects into the hydrophobic pocket formed by transmembrane (TM) domains 1a, 6b, and 7. The in-teraction of the benzene ring with the Phe254 residue in TM6b leads to ferroptosis. Additionally, derivatives of erastin, such as imidazolidinone erastin (IKE) and piperazine eras-tin (PE), also induce ferroptosis [47]. Due to erastin’s low water solubility and unstable metabolism in vivo, the introduction of a piperazine group improves its water solubility and stability. PE was shown to induce ferroptosis in HT-1080 cells and athymic nude mice, thereby inhibiting tumor growth [48]. Similarly, IKE was found to induce cellular ferrop-tosis in a diffuse large B-cell lymphoma (DLBCL) model by inhibiting system Xc^−^, leading to GSH depletion and lipid peroxidation, as demonstrated in a study by Yang et al. [49]. When cells were treated with erastin, system Xc^−^ was shown to be inhibited by the block-age of radiolabeled cystine uptake, confirming that erastin indeed targets system Xc^−^ [14]. Other inducers of ferroptosis include sorafenib, salazosulfapyridine (SAS), and glutamate. Sorafenib, a clinically approved anticancer drug, is commonly used to treat advanced can-cers such as renal cell carcinoma and hepatocellular carcinoma. Sorafenib specifically inhibits the function of system Xc^−^, thereby increasing the body’s sensitivity to ferroptosis [50]. In a study by Scott J. Dixon, sorafenib was found to induce ferroptosis, and this effect was counteracted by small molecule inhibitors of ferroptosis, such as deferoxamine (DFO), β-mercaptoethanol (β-ME), and ferrostatin-1 (Fer-1). The inhibitory effect of sorafenib on system Xc^−^ was confirmed to be dose-dependent using a glutamate release assay [50]. SAS, a salicylic acid derivative synthesized in the 1940s by linking sulfapyridine and 5-ami-nosalicylic acid via an azo bond, is traditionally used to treat Crohn’s disease and rheu-matoid arthritis. Recently, this old drug has found a new application as a potent inhibitor of system Xc^−^ [51]. In a study by PW Gout, it was found that the malignant progression of Nb2 lymphomas was associated with the active utilization of system Xc^−^ by macrophages. When SAS was administered in vivo and in vitro, it effectively inhibited the growth of cancer cells in both cases [52]. SAS specifically inhibits the uptake of cystine by system Xc^−^, thereby increasing the organism’s sensitivity to ferroptosis. Glutamate, one of the most abundant amino acids, is critical for normal physiological processes. For example, gluta-mate is the main component of excitatory neurotransmitters and serves as a precursor for the synthesis of γ-aminobutyric acid (GABA) [38]. Extracellular glutamate is exchanged 1:1 with intracellular cystine via system Xc^−^, so high levels of extracellular glutamate in-hibit the uptake of cystine, leading to lower levels of GSH, increased cellular oxidative stress, and heightened sensitivity to ferroptosis [53]. Glutamate induces excitotoxicity and oxidative stress in neuronal cells, phenomena that are closely associated with ferroptosis [14]. Additionally, the glutamate metabolite alpha-ketoglutarate (α-KG) contributes to the accumulation of lipid peroxides within cells [38]. Glutamate is converted to α-KG in the mitochondria by the action of glutamic-oxaloacetic transaminase 1 (GOT1) [54]. In a study by Cai et al., α-KG was found to increase intracellular ROS levels, an effect that could be reversed by Fer-1, an inhibitor of ferroptosis. The study also found that α-KG promotes ROS generation through malate dehydrogenase 1 (MDH1)-mediated conversion to 2-hy-droxyglutarate (2-HG) under acidic conditions, leading to cellular ferroptosis, DNA dam-age, and the upregulation of TP53 [55].

Additionally, GPX4 is a crucial component of the system Xc^−^/GPX4 axis. Previous studies have shown that cells with upregulated GPX4 expression exhibit greater resistance to ferroptosis, whereas cells with downregulated GPX4 expression are more sensitive to ferroptosis [56]. The second group of ferroptosis inducers includes RAS-selective lethal 3 (RSL3), ML162, ML210, and C18, which act directly on GPX4, inhibiting its activity. This inhibition reduces the cell’s antioxidant capacity, leading to the accumulation of lipid per-oxides and thereby inducing ferroptosis [48].

RSL3, identified during a small molecule compound screen conducted by Yang et al., was named for its increased lethality in the presence of oncogenic RAS. Another com-pound identified in the same screen was named RAS-selective lethal 5 (RSL5) [38]. In Yang’s study, metabolomics and chemical proteomics techniques revealed that RSL3 and erastin induce ferroptosis with similar features, such as ROS and iron dependence [48]. It was also found that caspase inhibitors did not effectively prevent cell death induced by RSL3 or RSL5, but DFO and vitamin E successfully reversed RSL3- and RSL5-induced cell death, confirming that these compounds induce iron-dependent cell death [56]. Further-more, in cells with knockdown of mitochondrial voltage-dependent anion channel 3 (VDAC3), sensitivity to erastin- and RSL5-induced cell death was reduced, whereas the lethality of RSL3 remained unaffected. This finding suggests that erastin and RSL5 induce ferroptosis via VDAC3, while RSL3 appears to induce ferroptosis through a VDAC3-in-dependent mechanism [56]. Additionally, Yang et al. in 2012 found that RSL3 did not induce lipid peroxidation in a manner dependent on GSH depletion [48]. The chloroacetamide moiety of RSL3 is an active component that targets enzymes with nucleophilic active sites, covalently interacting with selenocysteine at the active site of GPX4 [57]. The specific substrate for GPX4, 7α-cholesterol hydroperoxide (7α-cholesterol-OOH), was not reduced following RSL3 treatment, further demonstrating that RSL3 inhibits GPX4 activity [48]. In Hu’s study, pomelo peel essential oil (PPEO) was found to mitigate neurological damage after IS by attenuating ferroptosis, an effect counteracted by the ferroptosis inducer RSL3 [58]. ML162 and ML210 were identified through a high-throughput screening of approxi-mately 300,000 compounds from the National Institutes of Health Small Molecule Repository (NIH-MLSSMR) and were found to have nanomolar inhibitory potencies against HRASG12V-expressing cell lines [59]. In a study by Wang et al. in 2019, it was found that HT1080 cell death induced by ML162 and ML210 could be reversed by Fer-1 [60]. Bu-tylated hydroxytoluene (BHT), a potent antioxidant commonly used in oleochemicals and cosmetics to prevent free radical-mediated lipid peroxidation, was found to reverse ML162-induced ferroptosis in SH-SY5Y cells in a study by Parisa Faraji et al. [61]. In addi-tion, Chen et al. designed a novel GPX4 covalent inhibitor named C18, based on RSL3 and ML162. C18 was found to significantly inhibit GPX4 activity, exacerbate the accumulation of intracellular lipid peroxides, and strongly induce ferroptosis [62]. C18 retains the chlo-roacetone fragment and introduces an N-benzylaniline and diphenylamine skeleton, im-proving the druggability and pharmacokinetic profiles. C18 binds to Sec46 of GPX4, sig-nificantly inhibiting GPX4 function and inducing lipid peroxidation and ROS elevation, with a stronger effect than RSL3. However, this effect is reversed by Fer-1 [62].

Ferroptosis inducer 56 (FIN56) is a specific inducer of ferroptosis [63]. Kenichi Shi-mada et al. identified this compound among 56 non-apoptosis-dependent lethal com-pounds [64]. FIN56 induces ferroptosis through two distinct mechanisms: (1) promoting the degradation of GPX4 and (2) binding to and activating squalene synthase (SQS), which depletes CoQ10 [64]. Interestingly, compared to RSL3, FIN56 induces a slower accumula-tion of ROS within cells, but neither erastin nor RSL3 has as strong an effect on the abun-dance of GPX4 as FIN56 [64]. Moreover, FIN56 treatment does not decrease the transcript-tional level of GPX4, but instead, it increases it. Rather than inhibiting GPX4 protein syn-thesis, FIN56 induces the post-translational degradation of GPX4 [64]. In a study by Sun et al., the treatment of breast cancer (BC) cells with increasing concentrations of FIN56 for 72 h demonstrated high cytotoxicity, which was inhibited by α-tocopherol (α-TOH) and liproxstatin-1 (Lip-1). The overexpression of SLC7A11 also inhibited FIN56-induced cell death [65]. SQS is an enzyme that acts downstream of 3-hydroxy-3-methylglutaryl-coen-zyme A (HMG-CoA) reductase in the mevalonate pathway. SQS couples two farnesyl pyrophosphates (FPP) to form squalene, with FPP serving as the raw material for CoQ10 synthesis. FIN56 activates SQS, inhibits CoQ10 synthesis, and ultimately depletes CoQ10, increasing cellular sensitivity to ferroptosis [64].

FINO2 is a compound containing a 1,2-dioxolane structure, with the peroxide moiety in FINO2 being essential for inducing ferroptosis [63]. Unlike other ferroptosis inducers, FINO2 is a specific pro-oxidant molecule that oxidizes ferrous ions, thereby inducing ferroptosis. It is more likely to induce ferroptosis in cancer cells compared to non-malignant cells [66]. In a study by Rachel P. Abrams et al., it was found that iron is critical for FINO2-induced ferroptosis; FINO2 did not induce cell death after DFO treatment, but the external addition of iron, such as ferric ammonium citrate (FAC), accelerated cell death induced by FINO2 [66]. FINO2 also induces lipid peroxidation, and the addition of arachidonic acid, a substrate for the lipid peroxidation process, increased the potency of FINO2. How-ever, the use of lipophilic antioxidants such as Fer-1 and Lip-1, which inhibit ferroptosis, as well as the broad-spectrum lipoxygenase inhibitor nordihydroguaiaretic acid (NDGA), inhibited FINO2-induced cell death [66]. In a study by Michael M. Gaschler et al., it was similarly found that FINO2 induced HT1080 cell death, which was inhibited by Fer-1. However, the apoptosis inhibitor zVAD-FMK did not prevent cell death, nor did necrostatin-1, an inhibitor of necroptosis, prevent FINO2-induced cell death [67].

#### 3.3.2. Inhibitors of Ferroptosis

Fer-1 was first discovered in 2012 by Scott J. Dixon et al. The researchers screened a lead compound library consisting of more than 9500 small molecules based on drug-like properties, solubility, and scaffold diversity, ultimately identifying Fer-1 as a potent inhibitor of ferropto-sis [14]. Fer-1 was shown to be effective in reversing ferroptosis in HT-1080 cells induced by erastin. Fer-1 interacts with lipid membranes through its lipophilic N-cyclohexyl group. In the Fer-1 analog SRS8-72, the N-cyclohexyl group is replaced by an N-cyclopropyl group, which retains some of its antioxidant capacity [14]. However, replacing the primary aromatic amine in the structure with a nitro group disrupts the antioxidant capacity of Fer-1 and its ability to reverse ferroptosis. This suggests that the primary aromatic amines may be linked to the free radical scavenging capability of Fer-1 [14]. In a study by Giovanni Miotto et al., it was found that the intrinsic mechanism by which Fer-1 inhibits ferroptosis involves scavenging alkoxyl radicals generated by ferrous iron in LOOHs. Interestingly, Fer-1 is not depleted in this pro-cess; instead, it reduces the alkoxyl radicals in a para-catalytic manner, with ferrous ions sim-ultaneously reducing the free radicals of Fer-1 and the ferrous ions in the activated iron pool [68]. In a study by Jun Chu et al., Fer-1 was shown to decrease ROS and MDA levels, reverse the reduction in SOD activity induced by glutamate, and elevate the expression of GPX4 in HT-22 cells [69].

Lip-1, α-tocopherol, and α-tocotrienols are known to scavenge lipid peroxidation and inhibit ferroptosis. Lip-1, a potent spiroquinoxalinamine derivative, was discovered through the high-throughput screening of small molecule libraries for potential ferropto-sis inhibitors using cellular assays [38]. Lip-1 reduces lipid peroxides by trapping peroxyl radicals and transferring hydrogen from the -NH group to the peroxyl radical [70]. It is highly effective at inhibiting ferroptosis even at low concentrations [70]. In a study by Jose Pedro Friedmann Angeli et al., Lip-1 was shown to inhibit lipid peroxidation in GPX4-deficient (GPX4^−^/^−^) cells. Importantly, Lip-1 does not inhibit other classical forms of cell death, such as tumor necrosis factor α (TNFα)-induced apoptosis, H_2_O_2_-induced cell ne-crosis, or TNFα/z-VAD-FMK (z)-induced necroptosis in the bona fide L929 model [71]. Additionally, Lip-1 treatment reversed RSL3-induced cellular ferroptosis and prolonged the survival of mice subjected to renal ischemia–reperfusion injury by inhibiting renal cellular ferroptosis [71]. In 1922, Herbert Evans and Katherine Bishop discovered a fat-soluble compound in fresh green leaves of lettuce, which they named vitamin E. This compound restored the reproductive ability of animals [72]. α-TOH, the main component of vitamin E, is a naturally occurring antioxidant found in foods such as almonds, hazelnuts, and peanuts. It has long been recognized as the primary antioxidant in biological membranes, scavenging peroxyl radicals and interrupting the chain reaction of lipid peroxidation [73]. Studies have shown that the intracellular distribution of vitamin E is directly proportional to the distribution of lipids, facilitating the scavenging of lipid free radicals by vitamin E [74]. Forty years ago, Lucy et al. proposed that the double bonds of the PUFA chains of phospholipids containing arachidonic acid form a pocket shape. The fourth and eighth methyl groups on the isoprenoid side chain of tocopherols fit into this pocket structure, forming a complex structure [75]. Later, Kanga et al. introduced the concept that α-TOH binds to unsaturated fatty acids, forming a complex in which the carboxyl group of the fatty acid and the hydroxyl group of α-TOH form a hydrogen bond, while the 9,10- and 12,13-cis double bonds of the fatty acid complement the methyl group on the chromanol moiety [75]. α-Tocotrienols, which can be obtained from the seeds of monocotyledonous plants and the fruits of dicotyledonous plants [73], have been shown in several studies to possess superior antioxidant capacity compared to α-TOH. Unlike α-TOH, to-cotrienols interact more effectively with lipid radicals due to their unsaturated isoprenoid side chains, resulting in an “arched” conformation on the chromanol ring [38,74]. Generally, the hydrogen on the branched chain reacts with free radicals in a redox reaction, reducing the accumulation of lipid peroxides and thereby decreasing cellular sensitivity to ferroptosis.

**Figure 3:**
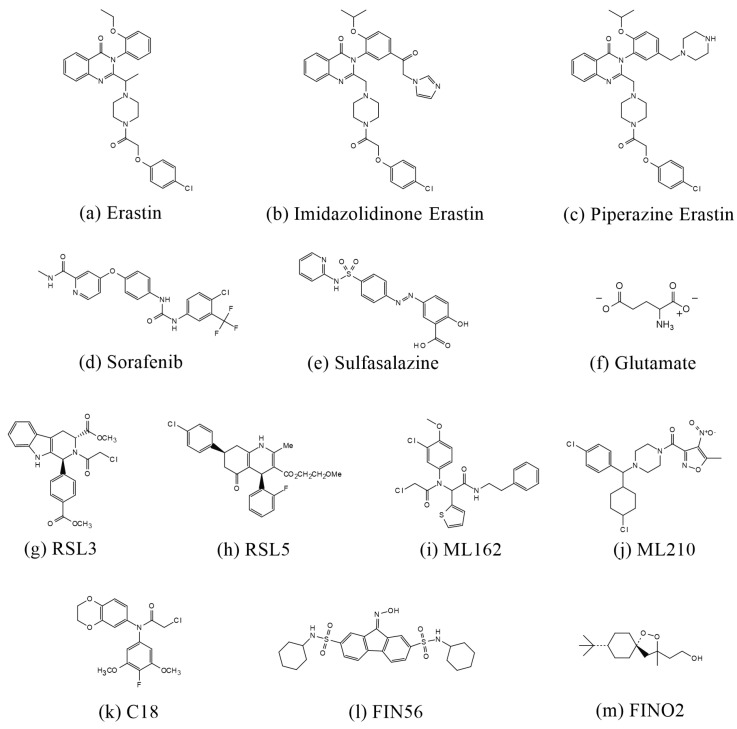
Structures of compounds that induce ferroptosis.

**Figure 4:**
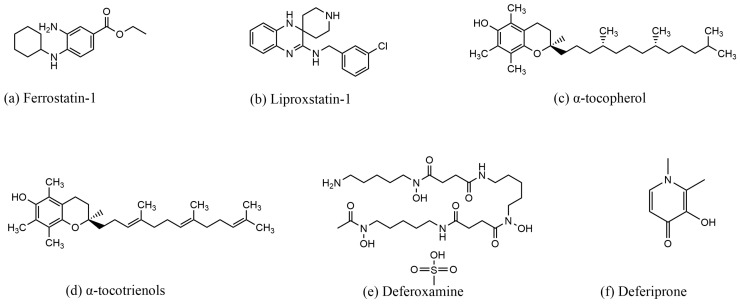
Structures of compounds that inhibit ferroptosis.

DFO and deferiprone (DFP, 1,2-dimethyl-3-hydroxy-4-pyridone) chelate Fe^3+^, reduce iron concentration, and inhibit ferroptosis. DFO is an iron chelator approved by the Food and Drug Administration (FDA) for the treatment of chronic iron overload [38,76]. First discovered in 1962, DFO is a colorless crystalline substance produced by *Streptomyces pi-losus*. It binds to Fe^3+^ in a 1:1 molar ratio and has a low affinity for other metal ions. Once bound to iron, the complex can be excreted through the kidneys [77]. In a study by Li et al., DFO reversed ferroptosis in ectopic endometrial stromal cells (EESCs) induced by erastin, a potent ferroptosis inducer [78]. Similarly, in a study by Guo et al., DFO reduced the levels of ROS, MDA, and Fe^2+^ in chondrocytes, thereby attenuating osteoarthritis (OA) through the nuclear factor erythroid 2–related factor 2 (Nrf2) pathway [79]. In another study by Yuan et al., DFO was shown to reverse sorafenib-induced ferroptosis in HSC-T6 cells while upregulating the expression of GPX4 and SLC7A11 proteins [80]. DFP, de-signed in 1985, is another iron chelator approved by the US FDA and the EU EMA for the treatment of iron overload in thalassemia [81]. DFP has a high affinity for iron ions and forms complexes with Fe^3+^ in a 3:1 molar ratio, although it can also bind to other metals such as copper, zinc, and aluminum [82]. DFP is capable of chelating iron from all intracellular iron pools, including low-molecular-weight iron, ferritin, and hemosiderin, as well as from transferrin and non-transferrin-bound iron (NTBI) in plasma [82]. Due to its high efficacy and low toxicity, DFP is used to treat a wide range of diseases, including neurodegenerative diseases, cardiovascular diseases, and conditions associated with free radical pathology [82]. In a study by Yao et al., DFP was shown to reduce the accumulation of total and ferrous iron in retinal ganglion cells induced by pathologically high intraocu-lar pressure (Ph-IOP). It also lowered MDA levels, elevated GSH content, increased GPX4 protein expression, decreased ACSL4 protein expression, and ultimately rescued cells from ferroptosis [83]. In another study by Hiroko Nobuta et al., DFP restored the activity of human PLP1G74E mutant oligodendrocytes, decreased the concentration of free extra-cellular iron, and reduced lipid peroxidation in cells [84]. The structure of the ferroptosis inducer are shown in Figure 3, the structure of the ferroptosis inhibitor are shown in Figure 4, and the mechanism of action of the ferroptosis inducer and ferroptosis inhibitor is shown in Table 1.

## 4. Advances in the Reduction in Ferroptosis by Chinese Herbs and Natural Products in the Treatment of Ischemic Stroke

rt-PA is the most commonly used treatment for IS. However, due to its narrow ther-apeutic window of 4.5 h and only moderate efficacy, as well as the risk of serious compli-cations, few patients are suitable candidates for this treatment. In recent years, the limita-tions of existing stroke treatment strategies have led to increased interest in herbal and natural medicines. Several studies have demonstrated that certain Chinese herbs and nat-ural products can treat IS by attenuating ferroptosis (Table 2 and Table 3).

### 4.1. Regulation of Ferroptosis by Nrf2

Nrf2 is a key regulator of oxidative stress in the body. Many studies have shown that traditional Chinese medicines (TCMs) can reduce ferroptosis and ameliorate the effects of IS by modulating Nrf2.

β-Caryophyllene (BCP), a naturally occurring bicyclic sesquiterpene, is found in foods and spices such as lemon, nutmeg, pepper, and clove [85]. Previous studies have shown that β-stigmasterol, in combination with the cannabinoid receptor II (CB2 receptor), exerts antioxidant and neuroprotective effects [86]. Recently, it was demonstrated that BCP improved neurological function scores, reduced infarct volume, and ameliorated related pathological features in middle cerebral artery occlusion reperfusion (MCAO/R) rats by enhancing Nrf2 nuclear translocation, activating the Nrf2/HO-1 pathway, and reducing oxygen-glucose deprivation and re-oxygenation (OGD/R)-induced ROS production and ferroptosis in primary astrocytes, thereby inhibiting ferroptosis [85].

In addition to BCP, several natural products have been found to exert anti-ferroptosis effects through the activation of the Nrf2 pathway, including Vitexin, Quercetin, and Rhein. Vitexin is a natural phytoflavonoid extracted from the leaves of Vitex cannabifolia, and is widely found in Vitex cannabifolia seeds, Vitex cannabifolia leaves, Phyllostachys nigra bamboo leaves, Hawthorn, and Passion Flower [87,88]. In a study by Guo et al., Vi-texin was shown to reduce the rate of Nrf2 translocation from the nucleus to the cytoplasm and modulate the Keap1/Nrf2/HO-1 signaling pathway to inhibit ferroptosis [88]. Quer-cetin, a naturally occurring flavonol compound, is one of the subtypes of flavonoids [89]. In a study by Yang et al., Quercetin was found to inhibit oxidative stress and protect the integrity of the BBB [90]. Previous studies have suggested that the antioxidant activity of Quercetin may be related to its ability to enter the cytoplasm, chelate iron, and reduce the pool of labile intracellular iron [91]. In a recent study by Peng et al., Quercetin was shown to improve neurological function, reduce infarct volume and pathological features, de-crease lipid peroxide accumulation, and inhibit ferroptosis by modulating the Nrf2/HO-1 signaling pathway in MCAO rats [92]. Although Quercetin is a promising protective agent in stroke, its poor solubility and bioavailability present challenges, and further research is needed to develop better dosage forms to enhance its therapeutic effects.

In a study by Liu et al., Rhein was shown to reduce cerebral infarct size and BBB damage, decrease oxidative stress and ROS production, and inhibit ferroptosis by mediating the Nrf2/SLC7A11/GPX4 signaling pathway [93]. Another natural product, Gastro-din, has also been reported to combat ferroptosis by activating the Nrf2 pathway. Previous studies demonstrated that Gastrodin improved cognitive dysfunction in vascular demen-tia rats by inhibiting ferroptosis through the modulation of the Nrf2/Keap1-GPX4 signal-ing pathway. Additionally, it can activate the Nrf2/HO-1 signaling pathway to inhibit fer-roptosis in HT-22 cells [94,95]. In a study by Zhang et al., the neutral polysaccharide of *Gastrodia elata* Blume (NPGE) was found to improve neurological function in MCAO/R mice by promoting Nrf2 nuclear translocation, activating Nrf2/HO-1, upregulating GPX4, decreasing the accumulation of ROS and Fe^2+^, and inhibiting ferroptosis [96].

Loureirin C, a dihydrochalcone extracted from dragon’s blood, was found to improve neurological function, reduce cerebral infarction, upregulate GPX4 protein, reduce ROS accumulation, and inhibit ferroptosis in MCAO/R mice through the activation of Nrf2 [97]. Icariside II (ICS II), derived from the TCM *Herba Epimedii* [98], was shown to promote Nrf2 dissociation from Keap1, increasing the transcriptional activity of Nrf2 [99]. Gao’s study further demonstrated that ICS II inhibited ferroptosis by activating Nrf2-mediated OXPHOS/NF-κB, reducing cerebral infarction, promoting long-term neurological func-tion recovery in mice, and attenuating the damage caused by OGD in astrocytes [98].

*Astragalus membranaceus* Bunge, widely used in the treatment of various diseases and recorded in Shen Nong’s *Herbal Classic* and *Compendium of Materia Medica* [100], contains Astragaloside IV (AST IV) as its main active ingredient. AST IV has been shown to protect against cerebral ischemia–reperfusion injury [100,101]. In a study by Wang et al., AST IV inhibited ferroptosis by activating Nrf2 transcription and modulating the P62/Keap1/Nrf2 signaling pathway [101]. Rehmannioside A was found to inhibit ferroptosis and exert neuroprotective effects by activating the Phosphoinositide 3-kinase (PI3K) /AKT/Nrf2 and SLC7A11/GPX4 signaling pathways. It also ameliorated cognitive impairment and neuro-logical deficits, reduced cerebral infarcts in MCAO rats, and decreased H_2_O_2_-induced tox-icity in SH-SY5Y cells, improving cell viability [102].

*Salvia miltiorrhiza* Bunge (SM), a plant from the Lamiaceae family, whose dried rhi-zomes are used medicinally, is widely distributed in China and Japan [103]. 15,16-Dihydrotanshinone (DHT), a lipophilic tanshinone extracted from SM, was found to reduce the accumulation of lipid peroxides and upregulate the expression of GPX4 proteins in PC12 cells, thereby inhibiting ferroptosis. It also ameliorated ferroptosis by decreasing the area of cerebral infarction through the activation of the Nrf2 pathway in PMCAO rats [104]. These findings provide valuable insights into the pathogenesis and potential therapeutic targets of IS.

### 4.2. Regulation of Ferroptosis by System Xc^−^/GPX4 Axis

The system Xc^−^/GPX4 axis is a crucial pathway for inhibiting ferroptosis, and several TCMs have been reported to exert their anti-ferroptosis effects via this axis.

Dihydromyricetin (DHM), extracted from various medicinal plants [105], has been shown to attenuate cerebral infarction, restore neurological function, and inhibit iron me-tabolism in MCAO/R rats. These effects are believed to be associated with the reduction in lipid peroxidation and iron levels, the upregulation of GPX4 expression, and the down-regulation of ACSL4 expression [106]. In a study by Li et al., baicalein reduced brain tissue iron levels and the accumulation of lipid peroxidation products in tMCAO mice. It inhib-ited ferroptosis by modulating the GPX4/ACSL4/ACSL3 pathway, thereby ameliorating cerebral post-ischemic reperfusion injury [107]. Galangin, a natural flavonoid extracted from the roots of *Alpinia officinarum* Hance, is also found in *Plantago major* L. and *Scutellaria galericulata* L. In a study by Guan et al., galangin improved learning and memory functions, reduced lipid peroxide accumulation, and inhibited ferroptosis in MCAO/R gerbils by mediating the SLC7A11/GPX4 pathway [108].

In addition to these natural products, some herbal compounds have been widely re-ported to exert anti-ferroptotic effects by modulating the system Xc^−^/GPX4 axis. Nao-taifang (NTF), composed of four traditional Chinese herbal medicines (*Radix Astragali* (Huangqi), *Rhizoma Chuanxiong* (Chuangxiong), *Pheretima* (Dilong), and *Bombyx Batryti-catus* (Jiangcan)), has been shown to improve neurological function in patients with acute cerebral ischemia [109]. Recent studies have demonstrated that NTF extract inhibits neu-ronal ferroptosis, reduces ROS, MDA, and iron accumulation, increases GSH levels, and improves neurological function in MCAO rats by modulating the TFR1/DMT1 and SLC7A11/GPX4 pathways [109]. Another Chinese herbal compound, Danlou tablets (DLT), was reported by Liu et al. to restore the integrity of the BBB, attenuate oxidative stress, and reduce cerebral infarction in tMCAO rats. DLT was also found to upregulate the pro-tein levels of SLC7A11 and GPX4, thereby inhibiting ferroptosis [110]. However, the active molecules responsible for regulating ferroptosis in DLT remain unidentified, and no sep-arate functional validation of the key active compounds in DLT has been performed. Fur-thermore, the long-term effects on tMCAO mice have not been studied.

Xingnaojing Injection, a TCM used to treat cerebral ischemic injury, is composed of musk, Borneol, Radix Curcumae, and Fructus Gardenia [111]. In a study by Liu et al., Xingnaojing Injection was found to ameliorate cerebral infarction and neurological deficits, upregulate GPX4 protein, and inhibit ferroptosis in MCAO rats [111].

Danhong Injection (DHI), which consists of *Salvia miltiorrhiza* (Dan Shen) and *Cartha-mus tinctorius* (Hong Hua), has also shown promise. In a study by Zhan et al., DHI reduced cerebral infarct area and associated damage in the brains of permanent middle cerebral artery occlusion (pMCAO) mice. It also enhanced the viability of OGD-injured HT-22 cells and ameliorated neuronal ferroptosis by activating the Special AT-rich sequence-binding protein 1 (SATB1)/SLC7A11/Heme Oxygenase-1 (HO-1) pathway [112].

### 4.3. Regulation of Ferroptosis through the PI3K/AKT Pathway

In addition to these pathways, certain herbal medicines inhibit ferroptosis by activat-ing the PI3K/AKT pathway. Angong Niuhuang Wan (AGNHW), a TCM known for its detoxification, resuscitation, and anticonvulsant properties, was historically used to treat acute illnesses [113,114]. Modern pharmacological studies have found that AGNHW can restore neurological function, reduce cerebral infarcts, inhibit lipid peroxidation and Fe^2+^ accumulation, and ameliorate ferroptosis in MCAO/R rats by modulating the PPAR and PI3K/AKT signaling pathways [115].

Paeoniae Radix, one of the most renowned Chinese herbs, has been used for over 1200 years [116]. Recent evidence has highlighted that extracts of *Paeoniae Radix Rubra*, including albiflorin, paeoniflorin, benzoyl paeoniflorin, oleanolic acid, and hederagenin, can reduce cerebral infarcts, ameliorate neurological deficits, upregulate the expression of GPX4, FTH1, Beclin1, LC3II, and p-Akt in the hippocampus of rats, and inhibit ferroptosis [117].

Increasingly, studies have shown that the active ingredients in TCM or TCM com-pound preparations can improve IS by regulating ferroptosis. While their efficacy is note-worthy, some shortcomings remain in existing research, such as identifying which active ingredients in TCM compound preparations are therapeutically significant and determin-ing whether the active ingredients of TCM, which have primarily been tested in animal models, are effective in clinical applications.

**Table 2:** Natural products attenuating ischemic stroke injury by modulating ferroptosis.

Active Ingredient or Formula	Source	Functional Mechanism	Experimental Models
β-Caryophyllene	lemon, nutmeg, pepper, clove, etc.;	Nrf2/HO-1	MCAO/R in male SD rats and primary astrocytes treated with OGD/R [85]
Vitexin	leaves of Vitex cannabifolia; widely found in Vitex cannabifolia seeds, Vitex cannabifolia leaves, Phyllostachys nigra bamboo leaves, Pennisetum millet, chaste tree, Hawthorn, and Passion Flower, among others	Keap1/Nrf2/HO-1	MCAO/R in male SD rats and primary cortical neuron cells treated with OGD/R [88]
Quercetin	coriander, onion, forsythia, okra, etc.	Nrf2/HO-1	MCAO in male SD rats; H_2_O_2_ or erastin induce HT22 cell ferroptosis [92]
Rhein	Rheum palmatum L., Cassia tora L., Polygonum multiflorum Thunb., and Aloe barbadensis Miller	Nrf2/SLC7A11/GPX4	MCAO in male SD rats and HT22 cells treated with OGD/R [93]
Gastrodin	gastrodia elata Blume	Nrf2/Keap1/GPX4, Nrf2/HO-1	BCCAO to establish vascular dementia models in male SD rats; HT22 cells establish a cell model of hypoxia injury [94,95]
Neutral polysaccharide of gastrodia elata Blume	gastrodia elata Blume	Nrf2/HO-1	MCAO models in C57BL/6 J mice, HT22 cells treated with OGD/R [96]
Loureirin C	Dragon’s blood	Nrf2/GPX4	MCAO/R model in C57BL/6 mice, SH-SY5Y cells treated with OGD/R [97]
Icariside II (ICS II)	Herba Epimedii	Nrf2/OXPHOS/NF-κB	MCAO models in C57BL/6 male mice and primary astrocyte treated with OGD/R [98]
Astragaloside IV (AST IV)	Astragalus membranaceus Bunge	P62/Keap1/Nrf2	MCAO/R models in male SD mice and SH-SY5Y cells treated with erastin or OGD/R [101]
Rehmannioside A	Rehmannia glutinosa Libosch	PI3K/Akt/Nrf2, SLC7A11/GPX4	MCAO models in male SD mice; H_2_O_2_-induced oxidative stress damage in SH—SY5Y [102]
15,16-Dihydrotanshinone (DHT)	Salvia miltiorrhiza Bunge	Nrf2/GPX4	PC12 cells and pMCAO models in male SD mice [104]
Dihydromyricetin	Ampelopsis grossedentata (Chinese vine tea), Hovenia dulcis (Japanese raisin tree), and some pinus and Cedrus species	Regulating the Expression of GPX4, inhibiting the SPHK1/mTOR signaling pathway	MCAO/R in male SD rats and HT22 cells treated with OGD/R [106]
Baicalein	Scutellaria baicalensis Georgi	GPX4/ACSL4/ACSL3	HT22 cells treated with OGD/R and tMCAO models in C57BL/6 male mice [107]
Galangin	Alpinia officinarum Hance	SLC7A11/GPX4	Using bilateral common carotid artery ligation in male gerbils established a cerebral ischemia model; hippocampal neuron cells treated with OGD [108]

**Table 3:** Traditional Chinese medicines attenuating ischemic stroke injury by modulating ferroptosis.

Traditional Chinese Medicine	Active Ingredient or Formula	Functional Mechanism	Experimental Models
Naotaifang	Radix Astragali (Huangqi), Rhizoma chuanxiong (Chuangxiong), Pheretima (Dilong), and Bombyx batryticatus (Jiangcan)	TFR1/DMT1, SLC7A1/GPX4	MCAO model in SD rats [109]
Danlou Tablet	Trichosanthes kirilowii Maxim, Salvia miltiorrhiza Bunge, Ligusticum chuanxiong Hort, Allium macrostemon Bunge, Paeonia lactiflora Pall, Pueraria lobata (Willd.) Ohwi, Alisma plantago-aquatica L., Astragalus membranaceus (Fisch.) Bunge, Davallia mariesii T. Moore ex Baker, and Curcuma aeruginosa Roxb	SLC7A11/GPX4	tMCAO model in male C57BL/6 mice and hy926 cell line treated with OGD/R [110]
Xingnaojing Injection	Musk, Borneolum, Radix curcumae, and Fructusgardenia	Upregulating GPX4, FPN, and HO-1 expression and downregulating COX-2, TFR1, and DMT1 expression	MCAO model in male SD rats; SH-SY5Y human neuroblastoma cells establish a hypoxia cell model [111]
Danhong injection	Salvia miltiorrhiza (Dan Shen) and Carthamus tinctorius (Hong Hua)	SATB1/SLC7A11/HO-1	pMCAO model in C57BL/6 mice, and HT22 and primary cortical neuron cells treated with OGD [112]
Angong Niuhuang Wan	Calculus bovis, powder of Cornu bubali, Moschus, Margarita, Cinnabaris, Realgar, Coptis chinensis Franch., Scutellaria baicalensis Georgi, Gardenia jasminoides J. Ellis, Curcuma aromatica Salisb., and Borneolum synthcticum	PPAR and PI3K/Akt	MCAO/R and ICH models in SD rats; erastin induces PC 12 cell ferroptosis [115]
Paeoniae Radix	Extract of Paeoniae Radix Rubra	PI3K/Akt	MCAO models in male SD mice; H_2_O_2_-induced oxidative stress damage in HT22 cells [117]

## 5. Conclusions

Initially, ferroptosis was thought to be an incidental and catastrophic form of cell death. However, it is now recognized as a consequence of imbalances in various metabolic pathways and the failure of key ferroptosis-regulating systems. The discovery of ferroptosis has opened new avenues in disease research, and its clinical significance in the occurrence, progression, and treatment of diseases is gradually being recognized. Despite these advances, the study of ferroptosis is still in its early stages, and many unanswered questions remain. How is the relationship between ferroptosis, autophagy, and other forms of programmed cell death defined? Are different types of cell death mutually reinforcing or antagonistic? These questions require further investigation. Future research should focus on exploring the pathogenesis of ferroptosis and its role in brain injury, discovering safe and effective treatments, and laying the foundation for treating brain injuries through the regulation of ferroptosis.

## Figures and Tables

**Table 1:** Summary of ferroptosis inducers and inhibitor.

Classification	Drugs or Compounds	Functional Mechanism
Inducer	Erastin	Inhibits system Xc^−^, prevents cystine import, and reduces GSH levels [14,47]
	Imidazolidinone Erastin	Inhibits system Xc^−^, prevents cystine import, and reduces GSH levels [47]
	Piperazine Erastin	Inhibits system Xc^−^, prevents cystine import, and reduces GSH levels [47,48,49]
	Sorafenib	Inhibits system Xc^−^, prevents cystine import, and reduces GSH levels [50]
	Salazosulfapyridine	Inhibits system Xc^−^, prevents cystine import, and reduces GSH levels [51,52]
	Glutamate	Inhibits system Xc^−^, prevents cystine import, and reduces GSH levels [14,38,53]
	RSL3	Binding to GPX4 leads to GPX4 inactivation [38,48,57]
	RSL5	Regulates iron accumulation by VADCS and promotes lipid peroxide accumulation [38,48,56]
	ML162 and ML210	Binding to GPX4 leads to GPX4 inactivation [59,60,61]
	C18	Covalently binds to GPX4 and inhibits GPX4 activity [62]
	FIN56	Promotes GPX4 degradation, binds and activates SQS, and depletes coenzyme Q10 [63,64,65]
	FINO2	Oxidizes Fe^2+^ and promotes iron accumulation [63,66,67]
Inhibitor	Ferrostatin-1	Inhibits the production of lipid ROS [14,68,69]
	Liproxstatin-1	Scavenges lipid peroxides [38,70,71]
	α-tocopherol	Scavenges lipid peroxides [73,74,75]
	α-tocotrienols	Scavenges lipid peroxides [38,73,74]
	Deferoxamine	Chelates Fe^3+^ and reduces iron concentration [38,76,79,80]
	Deferiprone	Chelates Fe^3+^ and reduces iron concentration [81,82,83,84]

## Data Availability

Data are contained within the article.
